# Wheat fungal endophyte communities are inseparable from the host and influence plant development

**DOI:** 10.1128/mbio.02533-23

**Published:** 2023-12-22

**Authors:** Or Sharon, Naomi Kagan-Trushina, Amir Sharon

**Affiliations:** 1 School of Plant Sciences and Food Security, Faculty of Life Sciences, Tel Aviv University, Tel Aviv, Israel; 2 Institute for Cereal Crops Research, Faculty of Life Sciences, Tel Aviv University, Tel Aviv, Israel; Cornell University, Ithaca, New York, USA

**Keywords:** fungal endophytes, plant tissue culture, axenic plant, mycobiome, plant fitness, *Triticum aestivum* (wheat)

## Abstract

Plants harbor complex and highly diverse fungal endophyte communities (FECs), making it difficult to evaluate the functional role of individual taxa, subsets of the community, or the FEC as a whole. To reduce the complexity of this system, we aimed to produce fungi-null wheat (*Triticum aestivum*) plants. To this end, we treated seeds with heat and fungicides and generated plants from rescued embryos and callus tissue. A culture-based approach and reverse transcription PCR analysis were negative, indicating that all treatments produced plants apparently free of fungi. However, the analysis of DNA using digital droplet PCR and next-generation sequencing revealed that tissues from all treatments retained low levels but diversity-rich FECs. While the FECs varied in composition across treatments and tissues, they all included core taxa of the mycobiome. The reduced fungal biomass, along with the changes in FEC composition, negatively affected plant development, supporting a FEC contribution to proper plant development and fitness. Our discovery that a large part of the FEC cannot be separated from plants and can be transmitted through seeds and tissue culture calls for reevaluation of particular microbiome paradigms, such as core taxa concepts, transmission modes, and functional species.

**IMPORTANCE:**

The native microbiome in a given plant must be considered when evaluating the effect of a single taxon or synthetic community. The pre-existing microbiome can interact with artificially added microbial cargo, which affects the final outcome. Such issues can be at least partially solved by the use of endophyte-free plants, which provide a clean background that should be useful in determining the effect of a single taxon, taxa combinations, or the entire microbiome on plant performance. Previous reports regarded plants as endophyte-free or axenic by the lack of fungal growth on culture media or the generation of plants from tissue cultures. We showed here that while fungi could not be isolated from fungicide-treated or tissue culture-regenerated plants, nevertheless, all plants contained rich fungal endophyte communities; namely, it was impossible to create fungi-free wheat plants. Our results call for rethinking fundamental microbiome-related concepts, such as core taxa, transmission mode, and functional species.

## INTRODUCTION

Fungal endophyte communities (FECs) are highly complex and dynamic, making it difficult to attribute specific effects to a single taxon or group of taxa. Furthermore, the composition of a given FEC is largely governed by interactions between taxa within the community (1, 2), complicating the identification of core and functional species.

Bread wheat FECs have been characterized in several studies (3–6). A recent meta-analysis of stem fungal endophytes in wheat and five cereal wild crop relatives showed that the core taxa in these plant species are composed of fungal taxa belonging to the genera *Cladosporium* and *Alternaria* (7). In addition, the yeast species *Candida sake* was found to be the most abundant taxon in wheat and was identified as the tier 1 core taxon. It is widely assumed that the core microbiome plays an important role in host plants. However, experimental evidence for the functional role of core species remains scarce (8).

One possible way to overcome some of these complications to aid in functional analyses of the core and other parts of the microbiome is to generate endophyte-free plants. Such plants can also aid in studies aimed to determine the effects of a single taxon or taxa combinations, facilitate microbial ecology studies, and serve as a tool in studies of microbe–microbe interactions within the plant microbiome. Methods to produce endophyte-free plants include heat and fungicide treatments. Heat has been used to remove endophytic *Epichloë amarillans* from its host *Agrostis hyemalis* (9) and produce endophytes-free wheat plants (10). Similarly, heat and fungicide treatments have been used to clean ryegrass (*Lolium perenne* and *Lolium multiflorum*) from Clavicipitaceous fungal endophytes (11). Various studies have reported that seed surface sterilization is sufficient to obtain endophyte-free seeds (12–15).

Tissue culture has also been attempted as a method to produce microbe-free plants. For example, strawberry and banana plants produced via the regeneration of meristems were reported to be fungi-free (16, 17), and wheat seedlings grown from sterile embryos were found to be bacteria-free (18). However, further studies reported the presence of fungi in embryos and calluses of different plant species (19–21), and the examination of common oak (*Quercus robur* L.) embryos using next-generation sequencing (NGS) revealed the presence of diverse fungal and bacterial communities (22).

Our original goal was to produce endophyte-free plants and seeds of bread wheat (*Triticum aestivum* L.) and use them to study the effect of fungal endophytes on wheat development. We used heat and fungicide treatments of seeds, production of plants from embryos and calluses, and combinations of all treatments. Surprisingly, we were unable to produce fungal-free plants; DNA quantification revealed dramatic reduction, but not elimination, of fungal biomass by all treatments, and NGS analysis demonstrated that the original tissues, resulting plants, and new seeds all contained FECs rich in fungal taxa and as diverse as the FECs in control plants. The new seeds with reduced fungal load exhibited lower germination rates and slower development. Collectively, our results show that fungal endophytes are an inseparable part of wheat plants and support an effect of fungal cargo quantity and composition on plant fitness and development.

## RESULTS

### Production of endophyte-free plants

#### Heat and fungicide treatments

First, we incubated seeds at 65°C for 30 min. There was no seed germination after 1 week, but about 20% of the seeds produced fungal cultures (Table S1). We next tested the effect of Sportak, a wide-range group 3 fungicide. Seed germination was unaffected at Sportak concentrations between 1% and 5%, yet yeast colonies were observed at all concentrations. When seeds were treated with Sportak without pre-incubation in water, germination rates dropped to less than 80%, and colonies of filamentous fungi developed in close to 40% of seeds (Table S1). Hence, heat or Sportak treatments were insufficient to eliminate seed fungal endophytes.

In search of a more effective treatment, we selected three additional fungicides (Table 1). We initially tested the sensitivity of five fungal species (*Mycosphaerella tassiana*, *Alternaria infectoria*, *Alternaria alternata*, *Cryptococcus magnus*, and *Cladosporium cladosporioides*) highly abundant in wheat FECs. The sensitivity of the different fungi to each of the fungicides varied, though all species were inhibited by a combination of the four fungicides (Table S2). Based on these results, we selected a treatment consisting of soaking the seeds for 2 h in a solution containing 1% of each fungicide. This protocol reduced seed germination to less than 50%, and fungal colonies developed in 6% of the seeds (Table S1). Germinating seeds before exposing them to the fungicides resulted in the survival of all seedlings and no apparent fungal growth, indicating that this procedure caused no harm to the seeds while preventing fungal development.

**TABLE 1:** Fungicides used in this study

Commercial name	Company	Active ingredient	Fungicide class
Sportak	Merhav Agro, Israel	Prochloraz	Class 3
Bayfidan 250	Lidor Elements, Israel	Triadimenol	Class 3
Folicur	Lidor Elements, Israel	Tebuconazole	Class 3
Ortiva Top	Adama, Israel	Difenoconazole + azoxystrobin	Classes 3 + 11

#### Plant regeneration

Plants were regenerated from both embryos and callus tissue; these plants were moved to sterile glass tubes with Murashige and Skoog (MS) medium and allowed to grow for 3 weeks. In both cases, no fungal growth was observed (Fig. S1).

**Fig 1:**
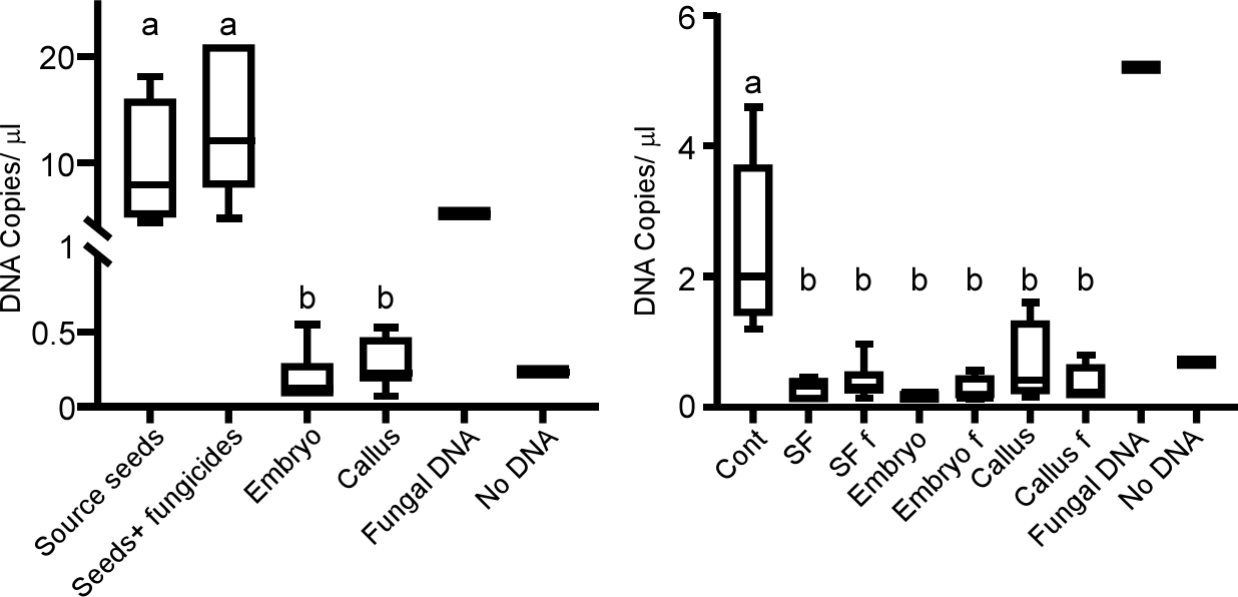
Amount of fungal DNA in source tissues and stems of the corresponding plants. Plants were produced in glass tubes with or without fungicides, samples were collected after 3 weeks, DNA was extracted, and the amount of fungal DNA was quantified via ddPCR using the fungal-specific ITS1F and ITS2 primers. “Fungal DNA” represented a positive control of DNA isolated from the fungi *Botrytis cinerea*. Left, copy number of fungal DNA in source tissues; right, copy number of fungal DNA in stems of the corresponding plants. Statistical differences between treatments were calculated using one-way ANOVA and Tukey *post hoc* test analyses. Treatments that are statistically different (*P* < 0.05) are represented by different letters.

To verify that the fungicide-treated and regenerated plants were indeed free of fungi, we produced seedlings using each of the aforementioned methods (Table 2), grew them for 3 weeks in sterile tubes with and without 0.1% fungicide mix (vol/vol) in the growing medium, and then sampled and plated plant tissues on potato dextrose agar (PDA). No fungi were detected in any of the treatments over a period of 30 days (Fig. S2A). We next extracted RNA from stem samples and performed reverse transcription PCR (RT-PCR) analysis using fungal-specific tubulin and wheat actin primers. The wheat actin primers produced a uniform band in all samples, while the fungal-specific primers produced an amplicon only in cDNA from stems of control plants and fungi (Fig. S2B). These results confirmed that fungi could not be isolated or detected in treated plants by culturing or RT-PCR analysis. To further verify that the plants were clean of fungi, we checked samples from all treatments by NGS. Unexpectedly, and in contrast to the isolation and RT-PCR analyses, the NGS data revealed that all plants contained fungal DNA (Table S4). Quantification by digital droplet PCR (ddPCR) showed at least a 10-fold reduction (copies/μL) in the amount of fungal DNA in embryos and calluses compared with seeds, as well as between the stems of control and treated plants (Fig. 1). These results demonstrated that the fungicide treatments and micropropagation did not eliminate the fungal endophytes in the treated plants and that the observed lack of fungal growth and RT-PCR amplicons was probably due to a drastic reduction in fungal load.

**TABLE 2:** Description of stem samples from different treatments

Treatment name	Treatment description
Cont	Stem samples grown from control seeds in glass tubes containing MS medium.
SF	Stem samples grown from seeds incubated in fungicides in glass tubes containing MS medium.
SF f	Stem samples grown from seeds incubated in fungicides in glass tubes containing MS medium supplemented with fungicides.
Embryo	Stem samples grown from embryos in glass tubes containing MS medium.
Embryo f	Stem samples grown from embryos in glass tubes containing MS medium supplemented with fungicides.
Callus	Stem samples grown from callus in glass tubes containing MS medium.
Callus f	Stem samples grown from callus in glass tubes containing MS medium supplemented with fungicides.

### FEC composition

#### Seeds and tissue cultures

**Fig 2:**
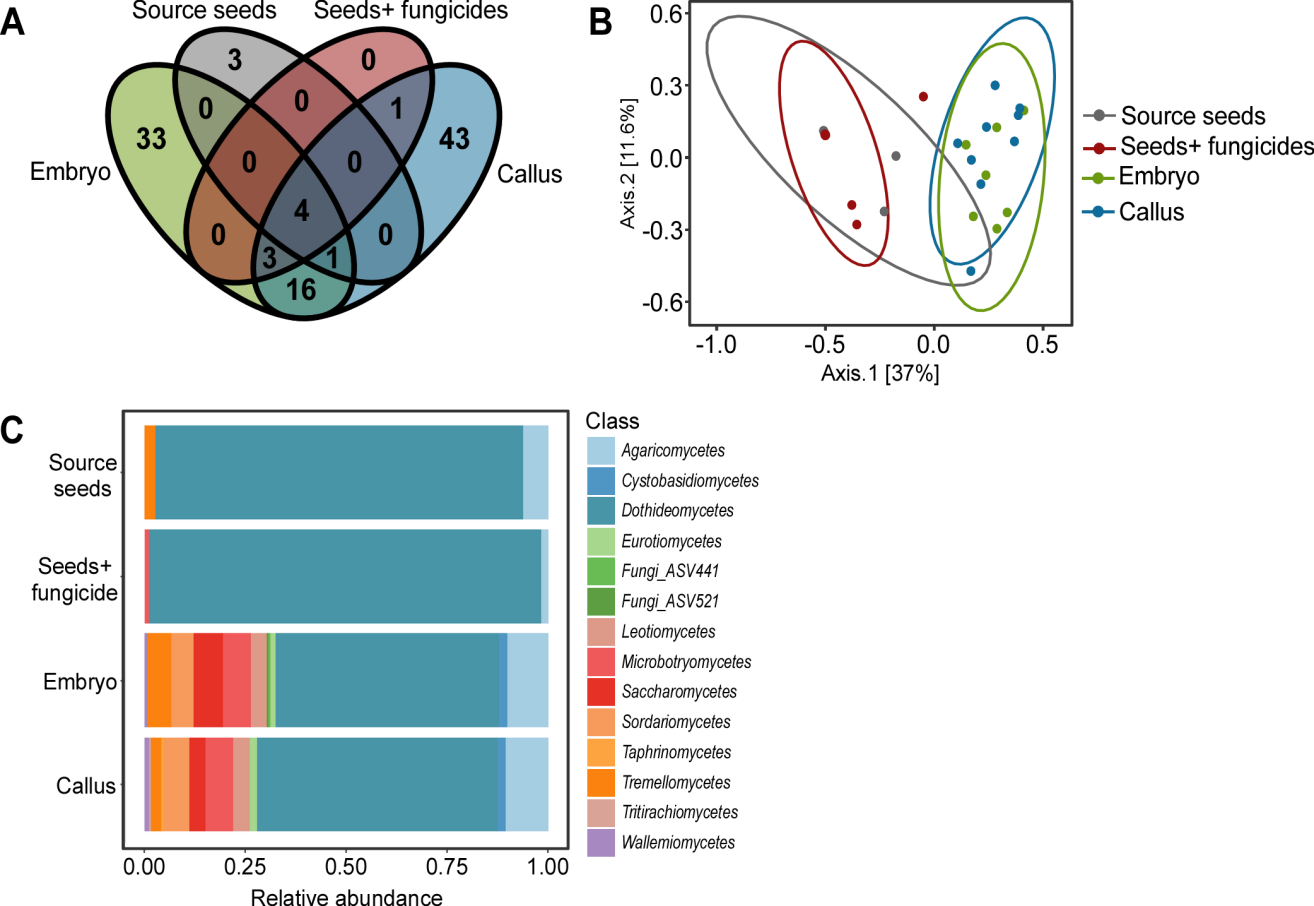
FECs in source tissues. (**A**) Venn diagram illustrating common and unique fungal taxa among the different tissues, (**B**) PCoA of FECs based on Bray–Curtis dissimilarity matrixes. Seed FECs are statistically different from embryo and callus FECs according to the PERMANOVA analysis (*P* < 0.001). (**C**) Class-level relative abundance of fungal taxa in the different source tissues based on Hellinger transformed abundance data.

Analyses of the amplicon sequence data revealed significant changes in the composition of the FECs in different tissues. The number of taxa was dramatically increased in embryos and calluses compared with seeds, from a total of 8 taxa in seeds to 57 and 68 taxa in embryos and calluses, respectively (Fig. 2A). In addition, taxa richness and Shannon index were four times higher in embryos and calluses (*P* < 0.001) (Table S5). Principal coordinate analysis (PCoA) of abundance using the Bray–Curtis dissimilarity matrix and permutational multivariate analysis of variance (PERMANOVA) analysis showed a clear separation between seed FECs and FECs of embryos and calluses (*F* = 4.7361, *R*^2^ = 0.38186, *P* < 0.001) (Fig. 2B; Table S6).

Changes in the FEC structure were also observed. *Dothideomycetes,* which predominated the FECs in seeds with a relative abundance close to 1 (0.97 and 0.99), decreased to an average of 0.5 in embryo and callus tissues, which in turn were enriched with yeasts and yeast-like classes, such as *Microbotryomycetes* and *Saccharomycetes* (Fig. 2C). Only 4 of the 104 taxa detected in the different tissues were common to all tissue samples: *Stemphylium ASV6*, *Coriolopsis gallica*, *A. infectoria*, and *A. alternata*, and the abundance of these taxa varied between different tissues (Table S7). Most significantly, *A. infectoria,* which was predominant in seeds (relative abundance of 0.7 in control and 0.84 in fungicide-treated seeds), dropped to less than 0.2 in calluses and embryos (Table S7). Unlike this predominance of *A. infectoria* in seeds, no single taxon was found to dominate the FECs in calluses and embryos, with the most dominant taxa in FECs from these tissues (*A. alternata* in embryos and *Cladosporium sphaerospermum* in calluses) having a relative abundance of 0.2 or lower. Consequently, the FECs of calluses and embryos had higher levels of evenness than seed FECs; Pielou’s evenness indexes in callus and embryo FECs were 0.94, compared to 0.36 and 0.4 in control and fungicide-treated seeds, respectively (Table S5). Other fungal taxa common to wheat, such as *Cladosporium* sp., were found in all samples except control seeds. These results demonstrate that wheat calluses and embryos are rich in fungal taxa and contain a large proportion of unique taxa that cannot be detected in seeds.

#### Treated plants

**Fig 3:**
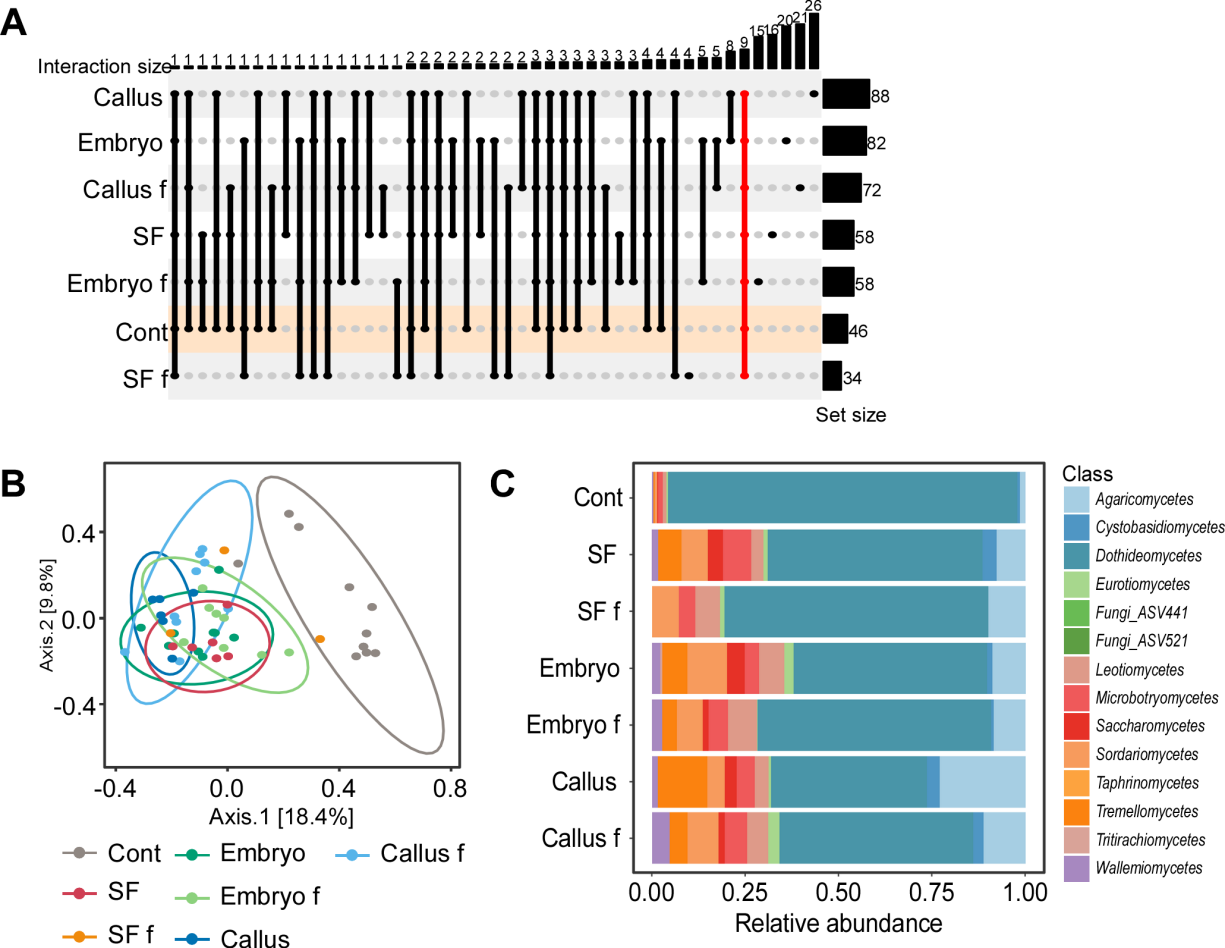
FECs in stems. (**A**) Upset plot illustrating common and unique fungal taxa among different stem groups. The line marked in red represents the number of taxa common to all treatments. Control samples are marked in orange. (**B**) PCoA of FECs based on Bray–Curtis dissimilarity matrixes. The FECs from control stems are statistically different from all the other FECs according to PERMANOVA and pairwise adonis analysis (*P* < 0.001). (**C**) Class-level relative abundance of fungal taxa in the different stems based on Hellinger transformed abundance data. Annotations for stem treatments can be found in Table 2.

Stems of plants regenerated from embryos and calluses contained more than 80 taxa, approximately twice the number in stems of plants from control (23) and fungicide-treated (24) seeds (Fig. 3A). Two of the DNA-free samples (negative controls) did not contain any reads and, therefore, did not pass the initial QA process using the QIIME2 platform. The third sample contained 1,772 reads with only three amplicon sequence variants (ASVs): *Botrytis cinerea*, *Botrytis caroliniana,* and *Stereum ostrea* (Table S7), indicating that the fungi found in the treated tissue were not due to contamination or false-positive results.

The differences in taxa richness between all groups were not statistically significant. FECs in stems from control seeds exhibited the lowest diversity (Shannon index) and were statistically different (*P* < 0.005) from the FECs of all other plant treatments (Table S5). PCoA using the Bray–Curtis dissimilarity matrix and PERMANOVA analyses showed a clear separation (*F* = 2.2237, *R*^2^ = 0.2326, *P* < 0.001) between the FECs from stems of control and treated plants (Fig. 3B; Table S6).

FECs in stems from treated seeds or regenerated plants had a different composition than FECs in stems of control plants. Similar to observed abundancies in source tissues, the relative abundance of taxa in the class *Dothideomycetes* dropped from 0.99 in stems of control plants to an average of 0.6 in stems of treated plants (Fig. 3C). *A. infectoria* proved to be the most abundant taxon in control stems (0.7) as well as stems from fungicide-treated seeds (0.4) and embryo-regenerated plants (0.35), but not in callus-regenerated plants (0.09), in which *M. tassiana* (0.26) and *A. alternata* (0.24) were the most abundant (Table S7). Pielou’s evenness index was found to be lower in control stems (0.4) and statistically distinct from treated stems (0.9), indicating that FECs in the stems of control plants are dominated by a small number of taxa, whereas those in stems of treated plants are evenly distributed. Although differences in taxa abundance were observed between the treatments, none proved statistically significant according to the Kruskal–Wallis statistical analysis.

In all treatments, stems of plants that grew in a medium supplemented with fungicides contained fewer taxa compared to those of corresponding plants grown in the fungicide-free medium (Fig. 3A). Additionally, the fungal taxa in stems of plants grown on the fungicide-supplemented medium differed from those in corresponding plants, i.e., the fungicides not only reduced the number of taxa but also affected taxa composition. For example, 82 taxa were found in stems generated from embryos, and this number dropped to 58 taxa when the medium was supplemented with fungicides, with only 25 taxa of the collective 115 taxa being common to both groups. Only 9 of the 200 taxa found in stems from all treatments were common to all groups: four *Cladosporium* sp., two *Alternaria* sp., *Blumeria graminis*, *M. tassiana*, and a single yeast taxon *Sporobolomyces roseus* (Fig. 3A).

Collectively, the NGS data show that changes in FECs in source tissues are extended to the corresponding stems and affect their FECs. They also demonstrate that supplementing the plant growth medium with fungicides reduces the number of taxa and changes the composition of the FECs in the corresponding plants.

### Analysis of new seeds

**Fig 4:**
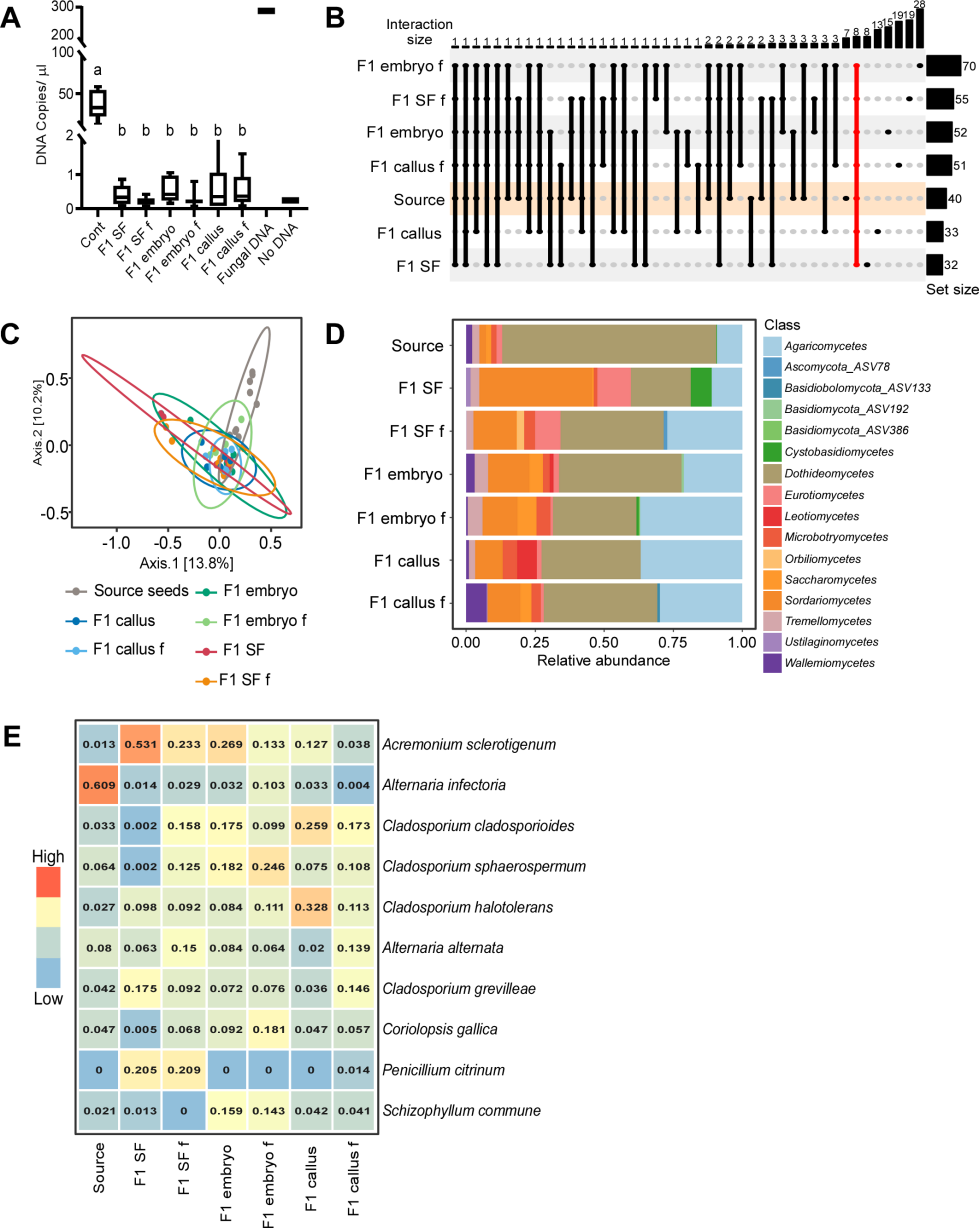
FECs in seeds. (**A**) Amount of fungal DNA in seeds. Plants were grown in a greenhouse, seeds were collected, surface sterilized, and DNA was extracted. The amount of fungal DNA was quantified by ddPCR using the fungal-specific ITS1F and ITS2 primers. Statistical differences between treatments were analyzed using one-way ANOVA and a Tukey *post hoc* test. Treatments that are statistically different (*P* < 0.05) are denoted by different letters. (**B**) Upset plot illustrating common and unique fungal taxa among different seed groups. The line marked in red represents the number of taxa common to all seed FECs. Source seeds (control samples) are marked in orange. (**C**) PCoA of FECs based on Bray–Curtis dissimilarity matrixes. The FECs from source seeds are statistically different from all the other new seed FECs according to PERMANOVA and pairwise adonis analysis (*P* < 0.001). (**D**) Class-level relative abundance of fungal taxa in seeds based on Hellinger transformed abundance data. (**E**) Relative abundance of the top 10 most abundant taxa in seeds. Values for each taxon represent the mean relative abundance. See Table 2 for details.

The reduced fungal biomass observed in stems of treated plants was extended to the new seeds; a drastic reduction of fungal DNA in seeds from the various treatments was evident compared to the source seeds (Fig. 4A). ITS amplicon sequencing on 70 seed samples (Tables S8 and S9) revealed 40 taxa in the source and new seeds; 32 and 33 taxa in seeds from fungicide-treated seeds and callus-regenerated plants, respectively; and up to 70 taxa in seeds of embryo-regenerated plants (Fig. 4B). Sequencing of negative controls resulted in no signal and was automatically removed in the QIIME2 QC process. No difference in FEC diversity was observed between the different groups of seeds (number of observed taxa and Shannon index) (Table S10), and the PCoA analysis based on the Bray–Curtis dissimilarity matrix did not separate FECs of the new seeds from FECs of the source seeds (Fig. 4C). However, PERMANOVA and pairwise adonis showed statistical differences between the FECs from new and source seeds, but not between the different groups of new seeds (*F* = 1.8605, *R*^2^ = 0. 0.20998, *P* < 0.001) (Table S11).

*Dothideomycetes* was the most abundant class within the entire seed FEC, but the relative abundance varied considerably between the different groups (Fig. 4D); it was highest in source seeds (0.91) and lowest (0.14) in seeds of plants produced from fungicide-treated seeds, in which *Sordariomycetes* was the most abundant class (0.53). *Agaricomycetes* yeasts were also enriched in new seeds from all treatments.

The FECs in the different treatments displayed high variability at the species level. Most significantly, *A. infectoria,* which had the highest relative abundance in source seeds (0.61), was reduced to below 0.05 in new seeds and was replaced by *Acremonium sclerotigenum* and *Cladosporium* sp. (Fig. 4E; Table S12). Taxa differential abundance between seed groups was not statistically significant according to the Kruskal–Wallis statistical analysis and Benjamini and Hochberg-adjusted *P*-value. Moreover, Pielou’s evenness index did not differ significantly between source and new seeds from treated plants (Table S10).

The number of fungal taxa varied between the different seed groups, from 70 taxa in seeds of plants produced from fungicide-treated embryos to 32 in seeds of plants produced from fungicides-treated seeds (Fig. 4B). Most of the taxa (109 out of 134 taxa) were unique to a specific group, and only eight taxa were common to seeds from all treatments: four *Cladosporium* sp., two *Alternaria* sp., *A. sclerotigenum*, and *C. gallica*.

Generally, the new seeds contained similar or fewer taxa compared to the stems of the progenitor plants. One hundred thirteen of the 167 fungal taxa in the new seeds were also found in the stems. A comparison of the FECs in each group of the new seeds to the FECs in stems of the corresponding plants showed that generally, 24% of the taxa were unique to the seeds, 28% were common to seeds and stems, and the rest of the taxa were found in one or more of the stem groups. Only four taxa were shared between seeds and stems from all treatments: *A. infectoria*, *A. alternata*, *C. sphaerospermum*, and *C. cladosporioides*.

Collectively, these analyses of the new seeds showed that despite constant exposure to fungicides and growth in clean conditions, the resulting seeds contained a low biomass but a rich diversity of FECs, accompanied by drastic changes in the composition of the FECs compared with the source seeds.

### Effect on plant fitness

To check whether these changes in fungal load and composition affect plant fitness and development, we compared the germination and growth of source seeds with those of new seeds from three treatments: (i) callus, where *Cladosporium halotolerans* was the most abundant taxon (0.33), (ii) fungicide-treated seeds, where *A. sclerotigenum* was most abundant (0.55), and (iii) fungicide-treated callus, which had no dominant taxon (Table S12).

**Fig 5:**
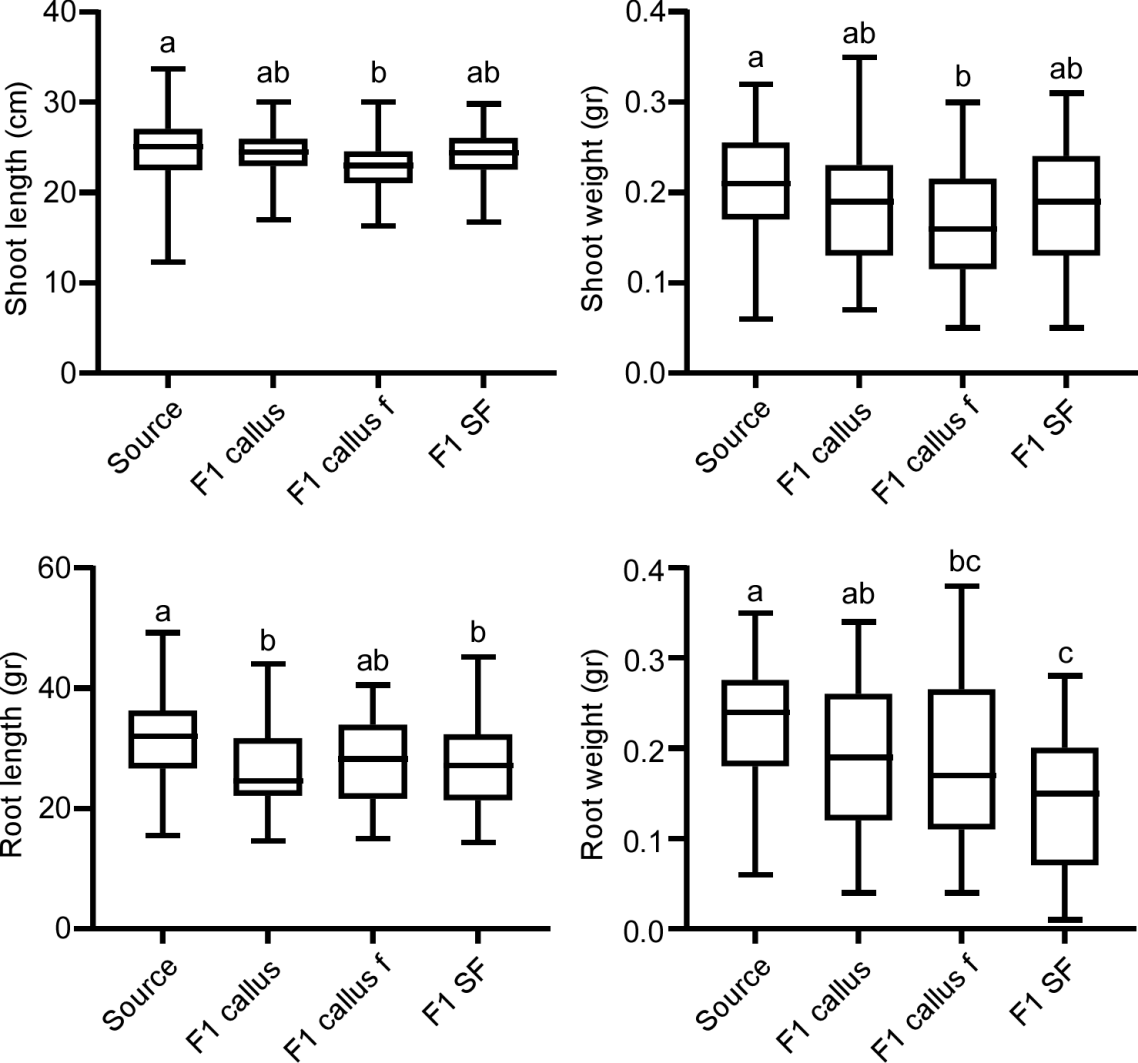
Effect of seed treatments on seedling biomass and size. Germinated seeds were planted in sand and grown in a clean greenhouse. After 14 days, the seedlings were pulled out of the soil and washed intensively, and the root and shoot length and biomass were recorded. Statistical differences were analyzed using one-way ANOVA and a Tukey *post hoc* test. Treatments that are statistically different (*P* < 0.05) are denoted by different letters. See Table 2 for details.

The source seeds had 98% germination rates, compared with an average of 82% in seeds from the different treatments (Fig. S3), a difference that did not prove to be statistically significant. Fourteen-day-old seedlings that developed from the new seeds had shorter shoots and roots and lower biomass than seedlings that developed from the source seeds. Among the three treatments, seedlings from fungicide-treated callus had the shortest shoot length (Fig. 5). Seeds of plants from callus and fungicide-treated seeds produced seedlings with shorter roots compared to controls, and seedlings from fungicide-treated seeds had the lowest root biomass compared to all other groups (Fig. 5). These results indicated that reduced fungal load and the observed specific changes in the composition of the FECs had a variety of negative effects on the early stages of plant development.

**Fig 6:**
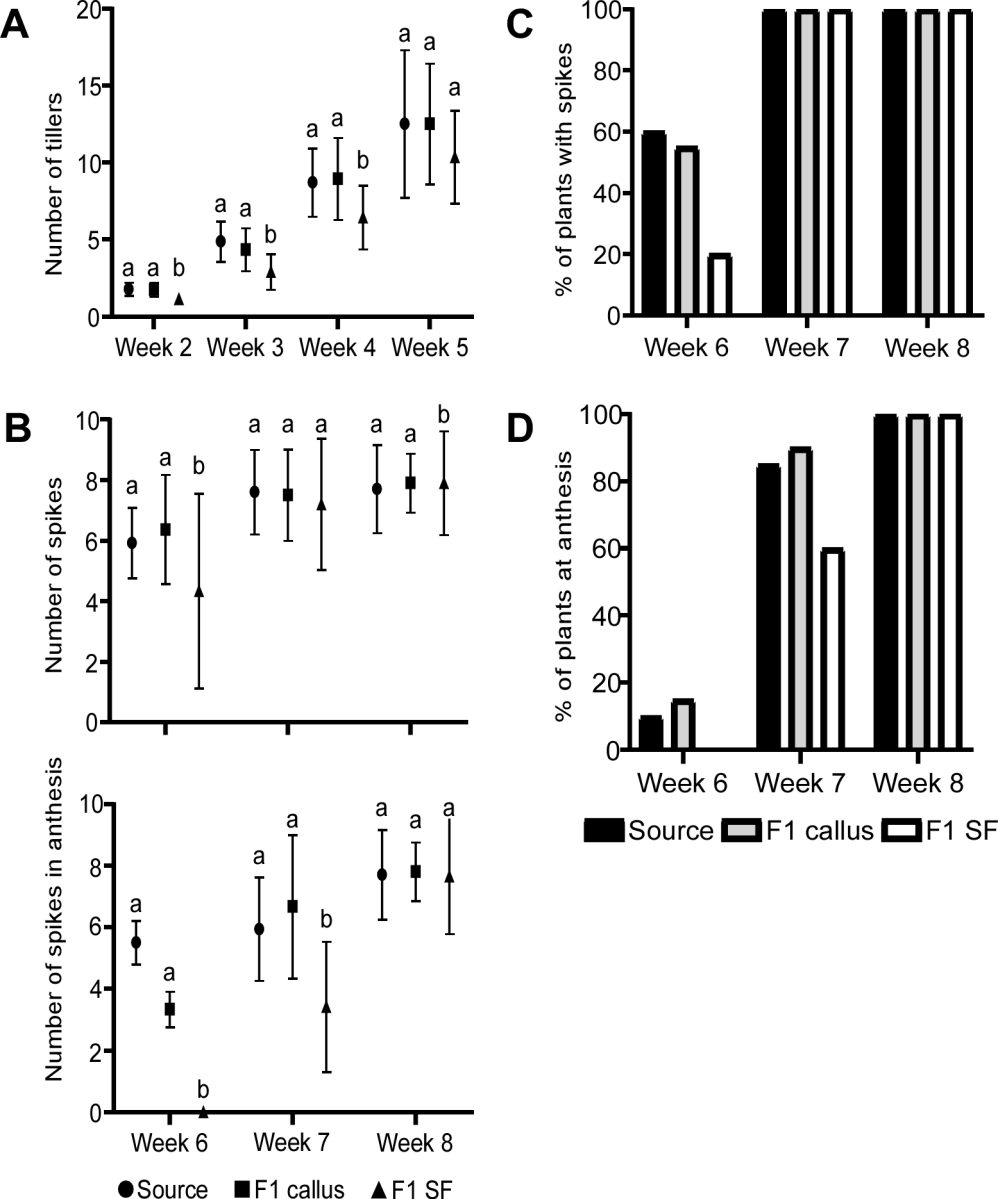
Effect of seed treatments on plant development. Germinated seeds were planted in 3 L pots with peat and grown in a clean greenhouse until maturation. Developmental parameters were recorded during the growth period: (**A**) number of tillers, (**B**) number of spikes and number of spikes at anthesis, (**C**) percentage of plants with spikes, and (**D**) percentage of plants with spikes during anthesis. Statistical differences between treatments were analyzed using one-way ANOVA and a Tukey *post hoc* test. Treatments that are statistically different (*P* < 0.05) are denoted by different letters. See Table 2 for details.

Based on the phenotype displayed in the previous experiments, we selected seeds of plants produced from fungicide-treated seeds and calluses and tested the effect of the changes in fungal endophyte composition and biomass on plant development from seedlings to maturation. Plants from fungicide-treated seeds showed the strongest developmental changes: they exhibited stunted growth (Fig. 6), the lowest number of tillers at 3 weeks, and the lowest shoot weight at 4 weeks (Fig. S4). At 6 weeks (heading), the plants of fungicide-treated seeds had a lower percentage of plants with spikes and fewer spikes in total than the other groups, and during the first 2 weeks of anthesis, they had the lowest number of flowering spikes (Fig. 6). The plants grown from seeds of callus-regenerated plants showed no statistical differences from control plants grown fron source seeds in any of the measured parameters. At maturation, there were no statistically significant differences in yield (number and weight of seeds) or in spike and stem weight between any of the treatments (Fig. S5).

Overall, reducing the fungal load had a generally negative impact on plant development. However, the data indicate that the specific changes in FEC composition resulting from different treatments had distinct effects on plant development that are potentially even more significant than changes in fungal biomass.

## DISCUSSION

The complexity of endophyte communities makes it difficult to evaluate the effects of individual taxa. To reduce the complexity of the system and simplify the analysis of specific members or portions of the FEC, we used previously reported approaches in an attempt to remove the native fungal cargo of wheat plants and generate fungal-free plants. While all treatments dramatically reduced the endophytic fungal biomass, in contrast to previous reports (10, 11, 16), plants retained a diverse FEC differing in amount and composition from the source FEC that could not be eliminated by any of the applied methods. These surprising results demonstrate an intimate and unbreakable relationship between plants and their FECs, including with species that are highly abundant in the environment and others that are potential pathogens. The reduction of fungal biomass had a negative effect on plant development, supporting the importance of the FEC for plant fitness.

A lack of visual evidence for fungal growth on plants or in a nutrient-rich culture medium has previously been taken as evidence for fungal-free plants (10, 25, 26), and working with sterile or axenic plants has been regarded as a straightforward practice (13, 14, 27). Our culture isolation and RT-PCR analyses are in line with these reports. However, as previously pointed out (21, 28), a lack of fungal growth, even after lengthy periods, is insufficient to determine whether the plants are indeed clean of fungi. We were able to find small amounts of fungal DNA in the different treatments using ddPCR (29). The signal in the negative control (no DNA) was not zero, the value representing the detection threshold. In some treatments, the level of fungal DNA was lower than this threshold, hinting at a lack of fungi in these tissues. However, the NGS data demonstrated that despite the drastic reduction in the amount of fungal biomass, all samples contain rich FECs.

Our NGS analyses showed that a wide range of fungi are transmittable through all stages of plant development, from seed to stem to seed, as well as in embryos and calluses. These taxa were not found in negative controls, and the community constantly shifted at each stage, from source tissue to stems to new seeds, excluding the possibility that they arose from external contaminations or false-positive results and confirming that the fungi are viable despite the lack of visible fungal growth and inability of RT-PCR to detect fungi in treated plants. Additionally, the newly developed plants were cultivated in aseptic conditions within sterile tubes for an extended period of time and, therefore, must have originated from fungi that were present in the original tissues. One possibility is that the detected fungi are metabolically inactive or dormant (30), which could explain their lack of growth and ability to survive the fungicide treatment. It is also possible that the host plant protects the fungi from the fungicides or that the fungicides do not reach the fungi at sufficient concentrations. Nevertheless, the lack of fungal growth despite the presence of viable fungi in the plant tissue is puzzling and requires further investigation.

All plant species carry FECs, which may include hundreds and even thousands of fungal variants (31–34). Certain groups in the FEC, such as mycorrhizae and endophytic *Epichloë* and *Serendipita* species, form mutualistic relationships that benefit their hosts. However, the vast majority of taxa are considered commensals without a particular functional role. Studies in wheat and related wild species have shown that the FECs include high proportions of low abundance sporadic taxa and a small subset of relatively stable and highly abundant taxa generally referred to as core taxa (3, 7, 24). It has been assumed that the core taxa are more closely associated with the plants and possibly have a functional role, either by a direct effect on the host (35, 36) or by shaping the assembly of the plant microbiome (37, 38). Our results showed not only that plants cannot be cured of fungi but also that the FEC retained within the plant includes a significant component of non-core and rare taxa. This finding adds to reports showing that a wide range of fungal endophytes, including saprophytic species and potential pathogens previously assumed sporadic or occasional, are capable of vertical transmission (4, 5, 22). It is important to note that the primers used in this study (ITS1f and ITS2) are biased toward certain fungal taxa and unable to detect fungi that lack the ITS region or have mutations at the primer sites (39). Furthermore, the ITS region lacks variability, which is needed to accurately identify species belonging to species-rich genera, such as *Fusarium* (40). Despite these limitations, we were able to detect a large number of rare taxa, and it is likely that more accurate taxonomical markers would have revealed an even greater community diversity. The discovery that a large part of the FEC is inseparable from plants and can be transmitted through seeds and tissue cultures is surprising and calls for reconsideration of certain microbiome paradigms, such as core taxa concepts, transmission modes, and functional species.

The presence of fungi within plant calluses and embryos has previously been reported. Calluses of *Bouteloua eriopoda* and *Atriplex canescens* were found to be encapsulated by fungi, though no fungi could be cultured from these tissues (21). NGS analysis conducted in an existing study demonstrated the presence of 21 fungal taxa in embryos of common oak (*Quercus robur* L.) (22), and 30 genera of bacteria were reported in embryos of eight wheat varieties (41). In accordance with these results, we detected 57 fungal taxa in embryos and 68 in calluses. The FECs in the tissue cultures and the corresponding regenerated plants contained all the most prevalent taxa of the wheat FEC (7), including *A. infectoria, A. alternata,* and *Cladosporium* spp., along with ASVs not previously detected in wheat seeds or stems, including plant pathogens such as *Pseudoidium neolycopersici* (tomato powdery mildew) and the obligatory wheat pathogen *Blumeria graminins*.

The FECs from different treatments differed in composition, and a large proportion of taxa were unique to each treatment. A few taxa were common to all treatments and can be considered core taxa as they are shared among several microbiomes of interest (35), in this case the source seeds and tissues, the new stems, and the new seeds. The most prominent common taxa that could not be removed from plants were *Alternaria* and *Cladosporium* species. These taxa have previously been found to be highly abundant in wheat and related crops and grasses (3–5, 24, 42, 43) and have been confirmed as core taxa in wheat and five wild grass relatives of cereal crops (7). While the *Alternaria* and *Cladosporium* taxa were detected in samples from all our treatments, their relative abundance differed considerably. *A. infectoria* was the most abundant taxon in the source seeds and was drastically reduced in the new seeds, unlike *Cladosporium* spp., which increased in embryos, calluses, and new seeds compared to the source seeds. These results are consistent with the reported relatively low vertical transmissibility of *Alternaria* sp. and the prevalent vertical transmission of *Cladosporium* sp. (4).

A primary goal of microbiome studies is to identify beneficial microbes (23, 44–46) or microbial consortia (47–50). When evaluating the effect of such single isolates or synthetic communities on plants, it is important to take the pre-existing microbiome into consideration. As expected, reducing the endophytic fungal load had a negative effect on plant development, but changes in FEC composition proved to be even more influential than the reduced biomass. For example, plants grown from seeds of fungicide-treated seeds exhibited delayed growth. The most noticeable impact of this treatment on FEC was the replacement of *A. infectoria* by *A. sclerotigenum* as the most abundant taxon. Both species are common endophytes in many plant species, including wheat (43, 51, 52), but also potential plant pathogens (53, 54). *Alternaria* species are the most highly abundant endophytes in wheat, though no beneficial effect has been demonstrated, while *A. sclerotigenum* has been reported as a potential biocontrol agent (51, 55) and was found to promote plant growth under water-limiting conditions in bread wheat (56). The strict classification of fungi according to a dominant endophytic or pathogenic lifestyle has changed in recent years; it is now more a rule than an exception that fungi change their lifestyles depending on conditions (57–59). The observed reduction in *A. infectoria* biomass may have impaired the balance within the FEC, resulting in an increased biomass of *A. sclerotigenum* that in turn led to a shift from a beneficial or commensal to a parasitic lifestyle.

Collectively, our findings show that changes in the composition and balance of taxa within the FEC can lead to changes in the effect on the host of specific FEC members, highlighting the need for a better understanding of the dynamics of microbe–plant and microbe–microbe interactions. While we were unable to cure plants of fungi, various aspects of microbiome research can benefit from the use of plants with reduced endophytes and modified FECs. Since greenhouse conditions have a profound effect on the composition of FECs (4), it will be important to evaluate the effects of different treatments or changes in FEC under natural conditions.

## MATERIALS AND METHODS

### Plant materials

Experiments were performed with seeds of wheat cv. Galil that were produced in 2015–2016 and stored in a dry (>15% humidity) storage room at 6°C.

### Heat treatment

The procedures were modified from protocols described by (11). Seeds were incubated for 4 h in a water bath at 23°C, dried, and then incubated for 30 min at 65°C. The treated seeds were placed in a Petri dish with PDA (Acumedia Manufacturers, Inc., Lansing, MI, USA). After 7 days, germination and fungal colony formation were recorded.

### Fungicide treatment

Ungerminated seeds were incubated at 23°C as per the heat-treated seeds, transferred for 1 h to a water solution containing 1%, 3%, or 5% (vol/vol) Sportak, and then placed on PDA. Seed germination and fungal colony formation were recorded after 7 days.

Germinated seeds (56) were incubated for 2–4 h in a water solution containing different combinations and concentrations of four fungicides (Table 1). The fungicide-treated seeds were transferred to sterile glass tubes with the half-strength MS medium and incubated in a growth chamber with a 16 h/8 h light–dark at 24°C in the light and 22°C in the dark. Plant survival and fungal colony formation were recorded after 3 weeks.

### Plant regeneration

Immature wheat seeds were surface-sterilized by soaking in 70% ethanol for 1 min, followed by 11% sodium hypochlorite for 11 min, rinsing five times with sterile double-distilled water (DDW), and the embryos were then dissected under aseptic conditions. See methods S1 for the detailed protocol.

### New seeds

Seedlings were produced in autoclaved glass tubes with the half-strength MS medium with or without 0.1% of the fungicide mix (vol/vol). After 3 weeks, the seedlings were moved into 0.5 L pots containing autoclaved sand. The plants were watered twice a week with sterile DDW supplemented with sterile Hoagland nutrient solution. Every 2 weeks, plants were treated with a mixture of fungicides. Spikes were covered with autoclaved paper bags before anthesis. All seeds were surface-sterilized by incubation in 3% bleach for 5 min, washed three times with sterile DDW, and then dried overnight in a laminar flow hood.

### Sensitivity of fungi to fungicides

We selected five fungal strains that represent highly abundant endophytes in wheat: *Mycosphaerella tassiana*, *Alternaria infectoria*, *Alternaria alternata*, *Cryptococcus magnus*, and *Cladosporium cladosporioides*. Mycelial plugs from 7-day-old colonies were transferred to 24-well plates with PDA supplemented by the tested fungicide combination and concentration. The plates were incubated at 22°C. Radial growth was recorded after 4 days. Each treatment included four replications, and the experiment was repeated three times.

### Fungal isolation

Three-week-old plants were cut into 3–5 cm sections and placed on plates with 10% PDA. Each plate contained leaves, shoots, and root samples from one plant. Fungal growth was monitored over a period of 30 days.

### Extraction of RNA and RT-PCR

RNA was extracted from 3-week-old stems using the TRIzol reagent (60). RNA samples (2 µg) were reverse-transcribed using a RevertAid First Strand cDNA Synthesis Kit (Thermo Scientific, USA). RT-PCR was carried out with two sets of primers: fungal tubulin primers BT2α/T222 (61, 62) and wheat-specific actin primers Actin_wheat_F (5′-AGGGAGTCCGTGAGATCCCGA-3′) and Actin_wheat_R (5′-ACCGTGCCCATTTACGAAGGAT-3′). See methods S1 for more details.

### DNA extraction and amplicon sequencing

Seed samples contained 1–3 seeds (100–150 mg); embryos from all the seeds in a single spike were pooled to create one individual sample (100–150 mg); a single undifferentiated callus and 100–150 mg of stem samples were used. Samples were placed in 2 mL sterile tubes, flash-frozen in liquid nitrogen, and stored at −80°C. Three negative controls (DNA-free) were produced from empty tubes that were otherwise processed in the same way as the rest of the samples. DNA was extracted from the empty tubes along with the true samples at three different time points. In addition, DNA was extracted in a separate lab in a hood dedicated to DNA extractions to avoid fungal and other contaminations. All samples were handled with autoclaved or filtered tools and reagents.

The samples were lyophilized overnight and ground with a Geno/Grinder 2000 (OPS Diagnostics, New Jersey), and DNA was extracted using a CTAB protocol (24). Fungal ITS amplicons, library preparation, and sequencing were performed according to reference (24). ITS1f (5′-CTTGGTCATTTAGAGGAAGTAA-3′) and ITS2r (5′-GCTGCGTTCTTCATCGATGC-3′) primers were used to create the amplicon libraries (63). Control seeds, fungicide-treated seeds, embryos, calluses, and stem samples were sequenced in one Illumina run; new seeds were sequenced in a separate run.

### Sequence processing and data analysis

Sequences were processed as described in reference (4) using the QIIME2 platform (64) and the DADA2 pipeline (65) with “Cutadapt” (66). Final data analysis was conducted in the R version 4.0.2 environment according to reference (4). See methods S1 for more details.

### Digital droplet PCR

Quantification of fungal DNA was performed with a QX200 Droplet Digital PCR System (Bio-Rad, Hercules, CA, USA). See methods S1 for more details.

### Plant phenotyping

Plant development was tested under optimal greenhouse conditions. For short-term experiments, plants were grown for 14 days from four different seed groups: source seeds, F1 SF, F1 callus, and F1 callus with fungicides. For long-term experiments, plants were grown from source seeds, F1 SF, and F1 callus seeds. For detailed experimental information, see methods S1 and Table 2.

## Data Availability

All sequences generated in this study have been deposited in the NCBI Small Read Archive (SRA) and are available under BioProject ID PRJNA904131. All codes used can be found at https://github.com/Or-Sharon/Sharon-et-al-2023.git

## SUPPLEMENTAL MATERIAL

Supplemental material 1 mbio.02533-23-s0001.docx Supplemental methods and figures.

Supplemental material 2 mbio.02533-23-s0002.xlsx Supplemental tables.

ASM does not own the copyrights to Supplemental Material that may be linked to, or accessed through, an article. The authors have granted ASM a non-exclusive, world-wide license to publish the Supplemental Material files. Please contact the corresponding author directly for reuse.
