# Supplementary material for: Wheat fungal endophyte communities are inseparable from the host and influence plant development
Source: mBio. 2023 Dec 22;15(2):e02533-23. doi: 10.1128/mbio.02533-23 (PMC10865843; doi:10.1128/mbio.02533-23)
Supplement: Supplemental material 1 — Supplemental methods and figures. [file mbio.02533-23-s0001.docx]

**Methods S1**

**Germination of wheat embryos**

Rescued immature embryos (14-21 days after anthesis) were placed on plates of MS medium (3 g/liter MS (M0222), 10 g/liter sucrose, 5.2 g/liter agar, pH 5.8). The embryos germinated in the dark for three days at 20–22 °C before being transferred to a growth chamber with a 16-h/8-h light-dark cycle at 24°C for the light and 22°C for the dark.

**Formation and regeneration of wheat callus tissues**

The protocol described by (1) was used with modifications. Immature wheat embryos were placed on WLS medium (4.3 g/liter Linsmaier & Skoog medium (LS; L0230, Duchefa, Haarlem, Netherlands), 40 g/liter maltose, 0.5 g/liter glutamine, 0.1 g/liter casein hydrolysate, 0.75 g/liter MgCl_2_۰6H_2_O, 1.95 g/liter MES, 2,4-D 0.00125 g/liter, picloram 0.0022 g/liter, ascorbic acid 0.1 g/liter, AgNO_3_ 0.85 g/liter, 2 g/liter phytagel, pH 5.8) without selection and incubated at 21°C in the dark until the formation of callus tissue. Initial calluses were transferred to LSZ medium (4.43 g/liter LS (L0230), 30 g/liter sucrose, 0.5 g/liter MES, 0.0025 g/liter CuSO_4_۰5H_2_O, zeatin 0.005 g/liter, 2 g/liter phytagel, pH 5.8) without selection and incubated at 24°C with 16 h light until roots were formed, then transferred to plates with MS medium (2.15 gr/liter MS (M0222), 0.5 gr/liter MES, 15 gr/liter sucrose, 2 gr/liter phytagel, pH 5.8) and incubated under the same conditions until seedling development.

**Growing plants under sterile conditions**

Germinated embryos, regenerated calluses, and fungicide treated seeds were grown for three weeks in autoclaved glass tubes with half strength MS medium with or without 0.1% of the fungicide mix. This concentration of fungicides was chosen as the concentration of 1% caused most of the seedling to die. Control plants were generated from surface sterilized and germinated wheat seeds grown on clean medium without fungicides.

**RT-PCR**

Reactions (25 μl) contained 0.7 μl of each primer (10 pmol), 12.5 μl Hay-Taq ready mix PCR reaction mix (HyLabs, Rehovot, Israel), 2 μl of template cDNA diluted 1:10, and 8.6 μl DDW. The fungal tubulin was amplified using the following program: denaturation at 96°C for 4 min, 29 amplification cycles at {96°C for 30 s, 55°C for 45 s, 72°C for 1 min}, and final elongation at 72°C for 10 min. Conditions for amplification of wheat actin were similar, with 35 cycles and annealing at 63°C for 30 seconds.

**Sequence processing**

Only the forward reads were used due to the lower quality of the reverse reads. Amplicon sequence variants (ASVs) were assigned taxonomical classification using the UNITE fungal reference (2) and the UNITE general FASTA release for eukaryotes using the Naïve Bayes approach with a minimum of 50 bootstraps. ASV agglomeration and filtration were done with the R package “phyloseq” (3). ASVs were agglomerated at the species level and filtered for rare taxa. Singletons, doubletons, and samples with less than 100 reads were removed from the dataset, and then taxa with relative abundance <0.005 (0.5%) were also removed.

**Bioinformatics analysis of the data**

The final data were transformed for relative abundance using Hellinger transformation (4). The following parameters—"Shannon Diversity Index," "Taxa Richness," and "Pielou's Evenness Index"—were used to measure alpha diversity using the “phyloseq” r-package. The differences in alpha diversity were statistically tested using a Kruskal-Wallis one-way analysis of variance followed by a pairwise Wilcoxon signed-rank test using the “rstatix” r-package (5). *P* values generated in the analysis were adjusted using the Benjamini and Hochberg method (6). Beta diversity was calculated with principal coordinates analysis (PCoA) using Bray–Curtis distances in the “phyloseq” r-package. To statistically test community composition differences, permutational analysis of variance (PERMANOVA) was calculated using the *adonis* function in the “vegan” r-package (7). *pairwise.adonis* function was used (8) to test statistical differences between community composition pairs following PERMANOVA analysis. To determine if differences in taxon abundance were statistically significant, we converted the loop t-test described in (9) to a Kruskal-Wallis test, followed by a pairwise Wilcoxon test. Graphs were generated using the “ggplot2” r-package (10). A Venn diagram was generated using the “ggvenn” r-package (11). Upset plots were created to illustrate shared and unique taxa in large numbers of sets (between groups of stems and new seeds) using the “ComplexHeatmap” r-package (12). A heatmap illustrating differences in taxa relative abundance was created using the “ampvis2” r-package (13).

**Digital droplet PCR (ddPCR)**

The ddPCR reactions contained 10μl of EvaGreen ddPCR Supermix (Bio-Rad, Pleasanton, CA, USA), 100nM of ITS1f and ITS2r primers (14), and 5ng plant DNA. 0.5 ng of *Botrytis cinerea* DNA was used as fungal positive control for the reaction. Cycling conditions included denaturation at 95°C for 10 min, followed by 39 cycles of {94°C for 30 s, 55°C for 1 min}, and a last step at 98°C for 10 min.

**Plant development experiments**

*Short term experiments with endophyte-reduced seeds.* In these experiments, plants were grown for 14 days. The experiments were performed with source seeds (control) and F1 seeds of plants that were produced from fungicide-treated seeds, calluses, and calluses growing on fungicide-supplemented medium. Seeds were germinated in petri dishes as previously described by (15) and germination rates were determined. Seedlings were planted in 0.2-liter pots containing autoclaved sand and grown in a greenhouse at 18°C–24°C with 14 h light cycle. The plants were watered twice a week. After two weeks, the plants were gently removed from the soil, thoroughly washed, dried, and shoot and root length and weight were measured. For shoot length the height of the plant from the root neck to the topmost leaf was measured. Root length was determined by the length of the longest root. The experiment was conducted three times, with 15 plants from each treatment.

*Long term experiments with endophyte-reduced seeds.* In this experiment, the plants were grown for three months. The experiments were performed with source seeds (control) and F1 seeds of plants that were produced from fungicide-treated seeds and from calluses. Seedlings were planted in 3-liter pots containing peat soil and grown in a greenhouse with 20°C–24°C temperatures and a 14 h light cycle. Depending on size and developmental stage, plants were watered once or twice a day until the end of grain feeling. Phenological parameters were recorded weekly throughout the experiment, including the number of tillers, spikes, and spikes in anthesis. Four weeks after planting, we sampled ten plants from each group and measured fresh and dry shoot weight. Mature and dried seeds were thrashed, counted, and their weight determined. The average seed weight was calculated as the product of the total seed weight and the number of seeds. The average number of seeds per spike was calculated as the number of seeds per plant divided by the number of spikes per plant. 30 plants were grown from each group.

*Statistical analysis*. All graphs and data generated from both experiments were produced using GraphPad Prism 8.0.2 for Windows (GraphPad Software, San Diego, California USA). One-way ANOVA followed by Tukey's test at 95% confidence level were used to test statistically significant differences between more than two treatments.


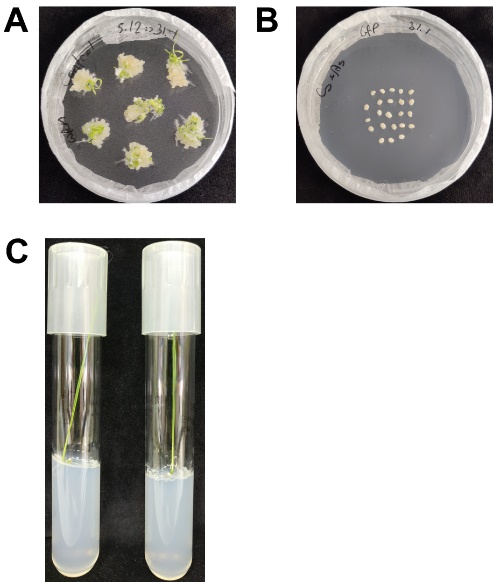


**Fig. S1** **Wheat tissue cultures.** (A) Callus tissue with shoots, (B) Immature embryos, (C) Glass tubes containing three-week-old plants that were regenerated from germinated embryos.


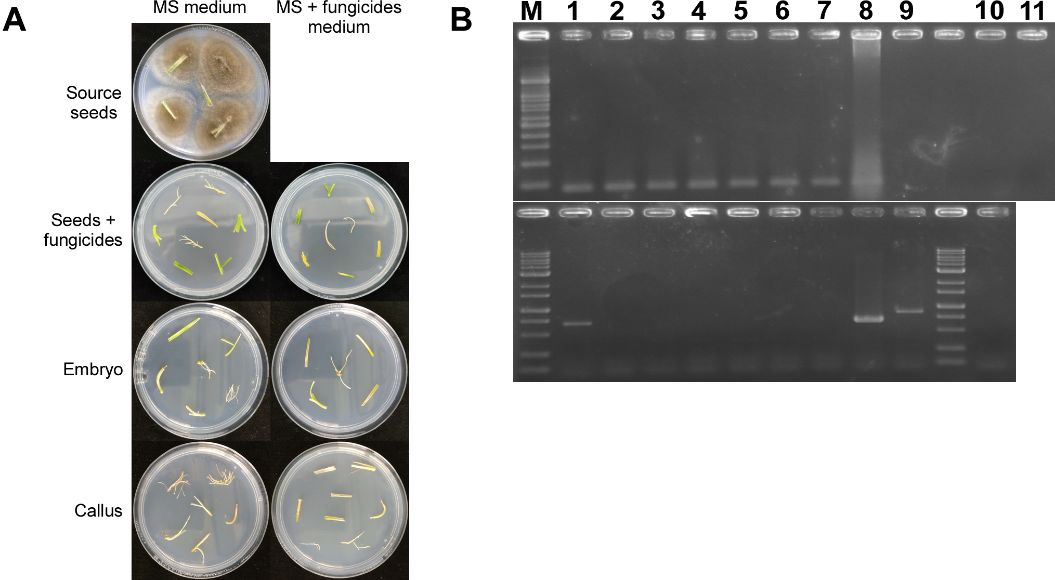


**Fig. S2 Analysis of treated plants by culturing and RT-PCR.** Plants were initiated from untreated source seeds (seeds), fungicide-treated seeds (seeds + fungicides), embryos, or calluses. (A) Plants were grown in glass tubes with MS for three weeks and then samples were collected, placed on PDA and development of colonies was monitored over 30 days. (B) RT-PCR analysis. Top – results of amplification of stems cDNA with primers for wheat actin (*ACT-1*). 1- Control seeds, 2- embryos, 3- embryos with fungicides, 4- fungicides-treated seeds, 5- fungicides-treated seeds grown on MS with fungicides, 6- calluses, 7- calluses with fungicides, 8- wheat DNA, 9- *Botrytis cinerea* cDNA, 10- *B. cinerea* DNA, 11- Reaction without template DNA. Bottom: results of amplification of stems cDNA with fungal tubulin primers (BT2α/T222). 1-7 as in the top panel, 8- *B. cinerea* cDNA, 9- *B. cinerea* DNA, 10- no DNA template.


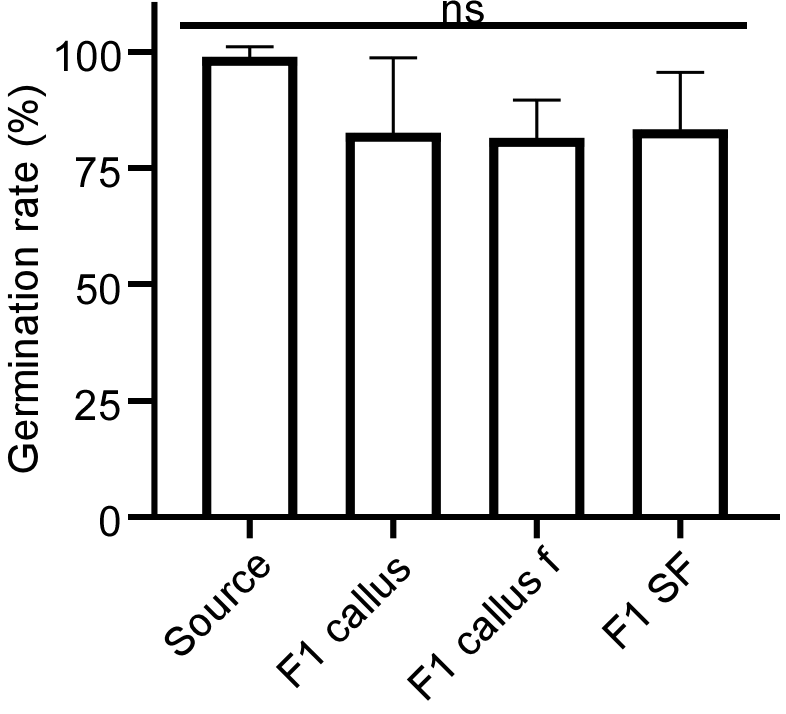


**Fig. S3 Seed germination rates.** Four seed groups were used in this experiment: source seeds and F1 seeds were collected from plants that were produced from calluses (F1 callus), calluses grown on fungicides (F1 callus f), and fungicide-treated seeds (F1 SF). The seeds were incubated on wet filter papers, and germination rates were determined after four days. The experiment was repeated three times, with a total of 155 seeds from each treatment. Average germination rates within the four treatments were statistically insignificant (*P* > 0.05) according to one-way ANOVA analysis and a Tukey post-hoc test.


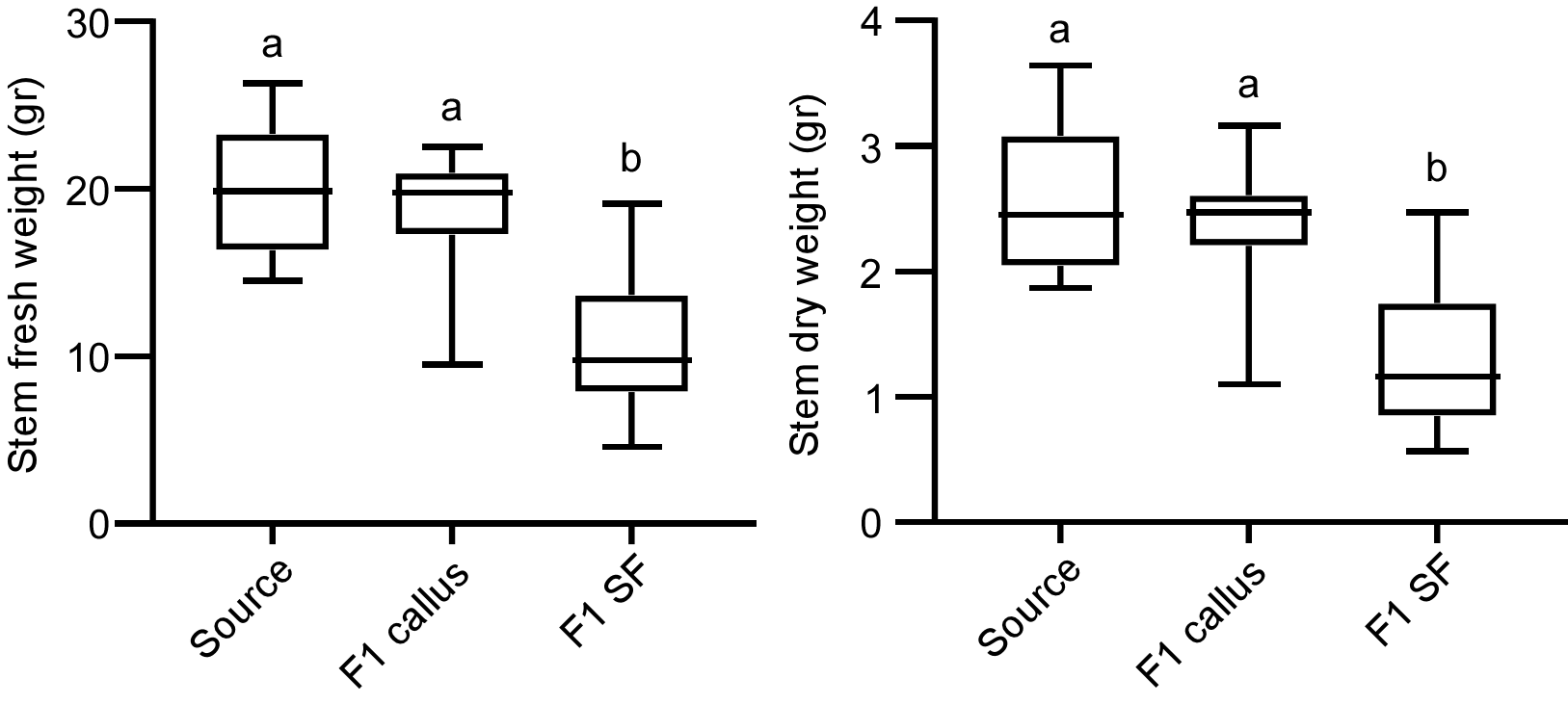


**Fig. S4**. Effect of seed source on plant development. Germinated seeds from three treatments (source, F1 callus and F1 SF) were grown in a greenhouse in 3-liter pots with peat soil under optimal conditions. Plants were sampled after one month of growth and the stems’ fresh (left) and dry (right) weight were recorded. Statistical differences were calculated using one-way ANOVA analysis and a Tukey post-hoc test (*P* < 0.05).


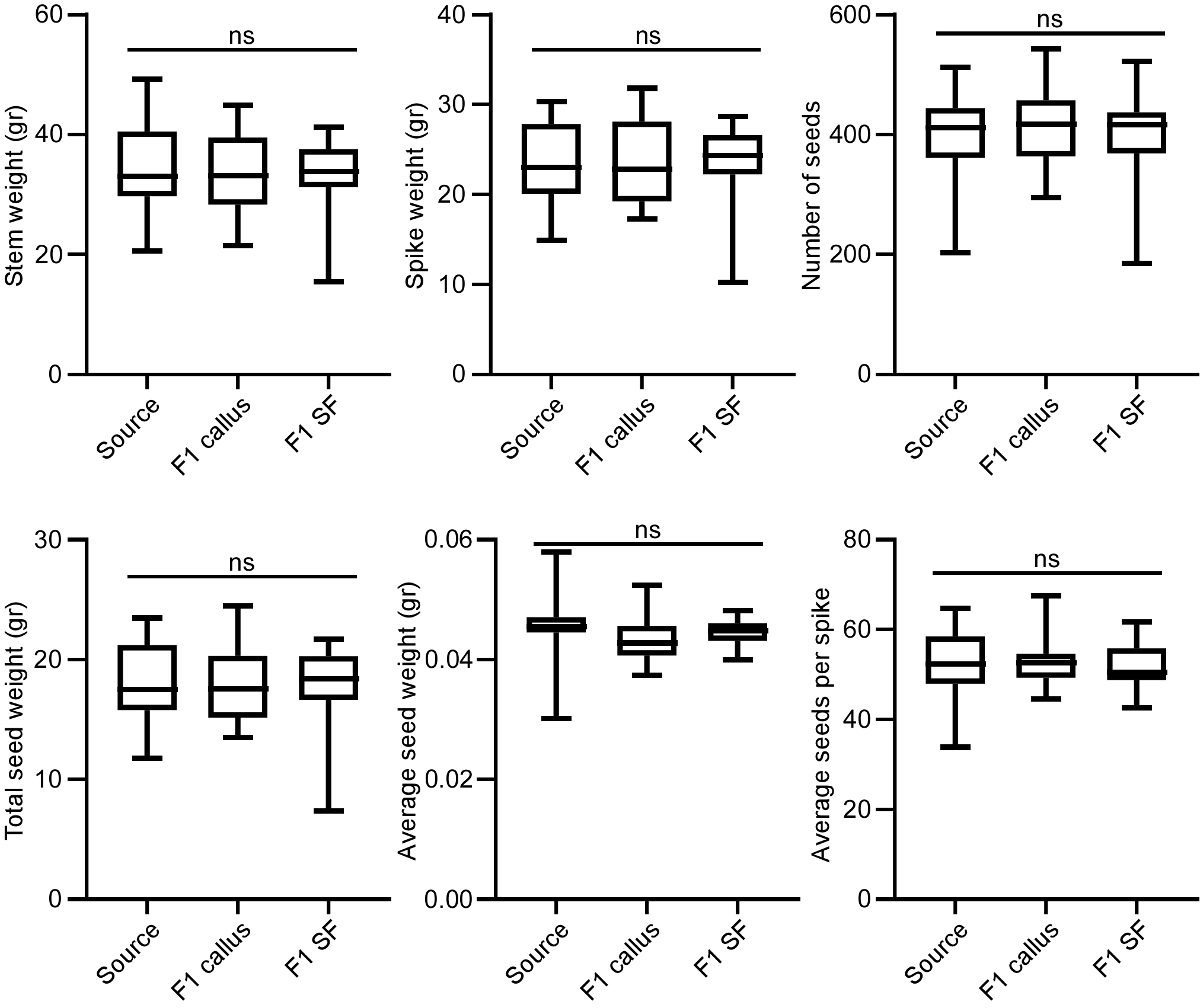


**Fig. S5 Effect of different seed source on yield.** Germinated seeds from three treatments (source, F1 callus and F1 SF) were grown in a greenhouse in 3-liter pots with peat soil under optimal conditions. Plants were sampled at maturation and stems weight, spikes weight, number of seeds, total seed weight per plant, average seed weight, and average number of seeds per spike were recorded. No statistical differences were observed in any of the parameters between the three treatments.

**References**

1. Ishida Y, Tsunashima M, Hiei Y, Komari T. 2015. Wheat (*Triticum aestivum* L.) Transformation using immature embryos, p. 189–198. *In* Agrobacterium Protocols. Humana Press Inc.

2. Nilsson RH, Larsson K-H, Taylor AFS, Bengtsson-Palme J, Jeppesen TS, Schigel D, Kennedy P, Picard K, Glöckner FO, Tedersoo L, Saar I, Kõljalg U, Abarenkov K. 2019. The UNITE database for molecular identification of fungi: handling dark taxa and parallel taxonomic classifications. *Nucleic Acids Res* 47:D259–D264.

3. McMurdie PJ, Holmes S. 2013. phyloseq: An R Package for reproducible interactive analysis and graphics of microbiome census data. *PLoS One* 8:e61217.

4. Legendre P, Gallagher ED. 2001. Ecologically meaningful transformations for ordination of species data. *Oecologia* 129:271–280.

5. Alboukadel Kassambara. 2021. rstatix: Pipe-friendly framework for basic statistical tests. https://cran.r-project.org/package=rstatix.

6. Benjamini Y, Hochberg Y. 1995. Controlling the false discovery rate: A practical and powerful approach to multiple testing. *J R Stat Soc Ser* B 57:289–300.

7. Oksanen J, Blanchet FG, Friendly M, Kindt R, Legendre P, McGlinn D, Minchin P, O’Hara RB, Simpson G, Solymos P, Stevens MHH, Szöcs E, Wagner H. 2020. vegan community ecology package version 2.5-7 November 2020. https://cran.r-project.org/web/packages/vegan/index.html.

8. Martinez Arbizu P. 2020. pairwiseAdonis: Pairwise multilevel comparison using adonis. R package version 0.4. https://github.com/pmartinezarbizu/pairwiseAdonis.

9. Wallen ZD. 2021. Comparison study of differential abundance testing methods using two large Parkinson disease gut microbiome datasets derived from 16S amplicon sequencing. *BMC Bioinformatics* 22:265.

10. Hadley Wickham. 2016. ggplot2: Elegant graphics for data analysis. Springer-Verlag New York. https://ggplot2.tidyverse.org.

11. Linlin Yan. 2021. ggvenn: Draw Venn Diagram by “ggplot2.” R package version 0.1.9. https://CRAN.R-project.org/package=ggvenn.

12. Gu Z, Eils R, Schlesner M. 2016. Complex heatmaps reveal patterns and correlations in multidimensional genomic data. *Bioinformatics* 32:2847–2849.

13. Andersen KS, Kirkegaard RH, Karst SM, Albertsen M. 2018. ampvis2: an R package to analyse and visualise 16S rRNA amplicon data. *bioRxiv* 299537.

14. Walters W, Hyde ER, Berg-Lyons D, Ackermann G, Humphrey G, Parada A, Gilbert JA, Jansson JK, Caporaso JG, Fuhrman JA, Apprill A, Knight R. 2016. Improved bacterial 16S rRNA gene (V4 and V4-5) and fungal internal transcribed spacer marker gene primers for microbial community surveys. *mSystems* 1:e00009-15.

15. Llorens E, Sharon O, Camañes G, García‐Agustín P, Sharon A. 2019. Endophytes from wild cereals protect wheat plants from drought by alteration of physiological responses of the plants to water stress. *Environ Microbiol* 21:3299–3312.
